# Vitamin D Decreases Risk of Breast Cancer in Premenopausal Women of Normal Weight in Subtropical Taiwan

**DOI:** 10.2188/jea.JE20100088

**Published:** 2011-03-05

**Authors:** Meei-Shyuan Lee, Yi-Chen Huang, Mark L Wahlqvist, Tsai-Yi Wu, Yu-Ching Chou, Mei-Hsuan Wu, Jyh-Cherng Yu, Chien-An Sun

**Affiliations:** 1 School of Public Health, National Defense Medical Center, Taipei, Taiwan (Republic of China); 2 Institute of Population Health Sciences, National Health Research Institutes, Miaoli, Taiwan (Republic of China); 3 Asia Pacific Health and Nutrition Centre, Monash Asia Institute, Monash University, Melbourne, Australia; 4 Graduate Institute of Medical Sciences, National Defense Medical Center, Taipei; 5 Department of Surgery, Tri-Service General Hospital, Taipei, Taiwan (Republic of China); 6 Department of Public Health, College of Medicine, Fu-Jen Catholic University, Taipei, Taiwan (Republic of China)

**Keywords:** vitamin D, menopause, BMI, sunlight exposure, subtropical

## Abstract

**Background:**

Evidence for an association between vitamin D status and breast cancer is now more convincing, but is uncertain in subtropical areas like Taiwan. This hospital-based case-control study examined the relationship of breast cancer with vitamin D intake and sunlight exposure.

**Methods:**

A total of 200 incident breast cancer cases in a Taipei hospital were matched with 200 controls by date of interview and menopausal status. Information on risk factors for breast cancer was collected in face-to-face interviews and assessed with reference to vitamin D intake (foods and nutrients) and sunlight exposure. Vitamin D intake was divided into quartiles, and threshold effect was evaluated by comparing Q2–Q4 with Q1.

**Results:**

After controlling for age, education, parity, hormone replacement therapy, body mass index (BMI), energy intake, menopausal status, and daily sunlight exposure, the risk of breast cancer in participants with a dietary vitamin D intake greater than 5 µg per day was significantly lower (odds ratio [OR], 0.48; 95% confidence interval [CI], 0.24–0.97) than that of participants with an intake less than 2 µg per day. In analysis stratified by menopausal status and BMI, both dietary vitamin D and total vitamin D intakes were associated with a protective effect among premenopausal women. There was a significant linear trend for breast cancer risk and dietary vitamin D intake in premenopausal women (*P* = 0.02). In participants with a BMI lower than 24 kg/m^2^ (ie, normal weight), dietary vitamin D intake was inversely related to breast cancer risk (*P* for trend = 0.002), and a threshold effect was apparent (Q2–Q4 vs Q1: OR, 0.46; 95% CI, 0.23–0.90).

**Conclusions:**

Vitamin D had a protective effect against breast cancer in premenopausal women of normal weight in subtropical Taiwan, especially an intake greater than 5 µg per day.

## INTRODUCTION

There are several known risk factors for breast cancer (BC), including alcohol intake,^1^ low body fat (in premenopausal women),^2^ positive energy balance,^3^^,^^4^ physical inactivity (in postmenopausal women),^5^ and height.^6^ Although Asian diets rich in soy products and phytoestrogens may offer some protection against BC,^7^ this is uncertain for hormone-sensitive BCs. Chinese diets and lifestyles may also be protective against BC, as breast cancer incidence is lower in Asians than in whites.^8^ Nevertheless, in Taiwan, BC incidence has tripled in 2 decades, and the age of highest risk is 10 years younger (40 to 49 years) than that in Western countries.^9^

Vitamin D plays a role in cell differentiation and may thus protect against BC.^10^^–^^13^ Ecological studies have suggested the possibility of such an effect,^14^^,^^15^ although there is uncertainty regarding the importance of menopausal status.^16^ In addition, no studies have addressed the association of vitamin D with BC in populations living at subtropical or tropical latitudes. This association may be relevant to the Chinese diet because of the known modulation of vitamin D receptor function by soy,^17^ the differences in foods containing vitamin D, and the role of skin synthesis of vitamin D with exposure to ultraviolet (UV) radiation.^18^ The vitamin D dietary reference intake (DRI) for adults in Taiwan is 5 µg per day (200 IU per day); however, little attention is paid to intake in Taiwan because it is presumed that UV light exposure provides adequate skin synthesis. There is a gradient in UV exposure from the subtropical north (the Tropic of Cancer runs through Taiwan at approximately latitude 25°) to the tropical south. In addition, UV exposure is affected by sunlight avoidance, which many Taiwanese prefer, as light skin is regarded as a sign of beauty.

The present study examined the associations of vitamin D intake and sunlight exposure with BC, with respect to menopausal status, fatness, soybean intake, and physical activity, in Taiwanese women whose habitual use of alcohol was negligible.

## METHODS

### Subjects

In this hospital-based, matched case-control study, cases and controls were women recruited from the Tri-Service General Hospital (TSGH) in Taipei, Taiwan during 2004–2005. Details of the study design have been described elsewhere.^19^^,^^20^ The cases were 200 women consecutively selected from patients who had received a first diagnosis of histopathologically confirmed BC at TSGH. Each case was matched by date of interview (±1 month) and menopausal status to a cancer-free control recruited from the TSGH Women’s Health Examination program. Eligible controls were women who had no cancer history and had undergone a 1-day comprehensive health checkup that included BC screening using X-ray mammography and ultrasonic examination. Over 90% of the women identified as potential controls participated in the study; they accounted for about 20% of all attendees. There were no significant differences in known BC risk factors between the included and excluded controls. We excluded subjects with metabolic disease and those who had resided in another country at least 1 year before the study. The Ethics Committee of TSGH approved the study.

### Data collection

Informed consent was obtained from all participants, who were interviewed in conformity with institutional guidelines for studies including human subjects. Information collected included sociodemographic characteristics, menstrual and reproductive history, menopausal status, lifestyle, and medical history, as well as family cancer history, and anthropometrics.

### Assessment of vitamin D intake

A 31-item semiquantitative food frequency questionnaire (FFQ) was administered to assess diet during the year before diagnosis (in cases) or the year before the interview (in controls), thereby disregarding recent changes in diet. The 7 intake frequency responses ranged from “never” to “6 or more times per day”. Dietary vitamin D intake was calculated from intakes of milk, dairy products, eggs, fish, seafood, organ meats, meat, and vegetables. Use of vitamin D supplements was also recorded. The vitamin D content was obtained from Japanese,^21^ US,^22^ and Taiwanese food industry data and from information on food labels. Total vitamin D intake was defined as the sum of vitamin D from food and supplements. The intra-class correlation coefficient of vitamin D intake from 65 study women was 0.9 for 2 repeats of the FFQ 1 month apart and 0.4 to 0.8 for various nutrients. Vitamin D intakes in the analysis were energy-adjusted, using the residuals approach.^23^

### Measurement of sunlight exposure

Subjects reported average daily sunlight exposure time during the prior year. Cross-checking questions asked about exposure during work and leisure time. Indirect exposure (through glass) under any conditions (sitting, driving, playing, or working) was not included. Time outdoors while using a parasol was not considered sunlight exposure. Sunscreen use in Taiwan was infrequent and not corrected for when calculating sunlight exposure. In total, 326 subjects provided information on sunlight exposure.

### Covariate classification and stratification

Confounder selection was based on a significant result in the present analyses or one reported in the literature. Because controls were mainly public servants who were younger and more educated than cases, we included age (<40, 40–45, 46–50, 51–55, 56–60, ≥60 years) and education (junior high school or below, senior high school, college), as well as parity (0, 1, 2, ≥3), hormone replacement therapy (HRT; yes, no), body mass index (BMI), sunlight exposure (<30, 30–59, ≥60 minutes per day), and total energy intake in the analyses. BMI (kg/m^2^) was classified as underweight (<18.5), normal (18.5–23.9), overweight (24–26.9), or obesity (≥27), using Taiwanese criteria.^24^ Adjustment was also made for plasma homocysteine because it is a putative risk factor for BC.^19^ Subjects were stratified by menopausal status and BMI due to the heterogeneity in BC incidence among these subpopulations.

### Statistical analysis

Conditional logistic regression or logistic regression (for sunlight exposure) was used to estimate odds ratios (ORs) and 95% confidence intervals (CIs) for BC risk associated with vitamin D intake. Multivariable models were adjusted for age, education, parity, HRT, BMI, energy intake, menopausal status, and daily sunlight exposure. To evaluate the linear trend, we entered the median of each quartile of vitamin D intake as a single continuous variable in the model and tested using the Wald test. Interaction between vitamin D intake (<5, ≥5 µg per day, adult DRI) and sunlight exposure (≤30, >30 minutes per day, median value) was examined. The combined effect of vitamin D intake and sunlight exposure was estimated by using low intake and low sunlight exposure as reference. To assess effect modification, analyses were stratified by menopausal status and BMI (<24 and ≥24).

## RESULTS

As compared with controls, cases were older (48.9 vs 52.5 years), less educated, had an older age at menarche, younger age at first full-term pregnancy, and higher parity; however, there was no difference in age at menopause. There were no differences in BMI, physical activity, family history of breast or ovarian cancer, use of HRT, nutrition supplement use, or sunlight exposure (Table 1).

**Table 1.** Characteristics of subjects^a,b^

	Cases (*n* = 200)	Controls (*n* = 200)
Age (y), mean (SD)^c^	52.5 (11.7)	48.9 (7.62)
<40	9.5^c^	6.0
40–49	38.0	51.0
50–59	28.5	34.5
≥60	24.0	8.5
Education^c^		
Some elementary school	26.5	13.0
Junior high school	12.5	8.5
Senior high school	29.0	36.5
Some college	32.0	42.0
BMI (kg/m^2^), mean (SD)	23.2 (3.26)	23.0 (2.99)
<18.5	4.5	3.0
18.5–23.9	59.5	64.5
24.0–26.9	21.5	22.0
≥27.0	14.5	10.5
Age at menarche (y), mean (SD)^c^	14.0 (1.71)	13.7 (1.53)
Age at menopause (y), mean (SD)	49.5 (5.24)	48.8 (4.71)
Age at birth of first child (y), mean (SD)^c^	25.8 (4.01)	26.7 (4.16)
<24	31.7	21.9
24–26	25.0	25.3
27–29	27.2	29.8
>29	16.1	23.0
Parity (times), mean (SD)^c^	2.53 (1.51)	2.00 (1.03)
0	10.0	11.0
1	7.5	12.5
2	38.5	49.5
≥3	44.0	27.0
Family history of breast cancer	4.5	3.0
Family history of ovarian cancer	1.5	1.0
Ever use of hormone replacement therapy	18.0	25.5
Frequency of physical activity		
Very low	45.9	41.4
<4 h/week	18.2	19.5
≥4 h/week	35.9	39.1
Sunlight exposure (minutes per day)		
<30	24.1	25.6
30–59	29.1	23.2
≥60	46.8	51.2
Use of any supplement	69.0	75.0
Use of vitamin D-related supplement	47.5	55.5
Use of vitamin D supplement	1.00	0
Homocysteine (nmol/mL)^c^	9.39 (4.21)	7.17 (2.03)

SD, standard deviation; BMI, body mass index.

^a^Values are percentages unless otherwise noted.

^b^Continuous variables were compared using conditional logistic regression analysis; categorical variable were compared using the chi-square test.

^c^*P* < 0.05.

Cases had a significantly lower daily energy-adjusted dietary vitamin D intake than did controls (3.30 µg vs 3.69 µg, *P* = 0.04), but total vitamin D intake (6.78 µg vs 7.33 µg, *P* = 0.10; Table 2) did not differ. There were no differences in the intakes of energy, soy products, or alcohol between cases and controls. Protein and calcium intakes were significantly lower in cases. After adjustment for potential confounders, women with higher protein intakes (in grams or as a percentage of total energy) had a significantly lower risk of BC. The OR for a 1% increase in total energy from protein was 0.86 (95% CI, 0.78–0.94).

**Table 2.** Daily intakes of nutrients and foods in cases and controls

	Cases (*n* = 200)		Controls (*n* = 200)		*P* value^a^	OR (95% CI)
		
	Mean	SD	Mean	SD		
Total vitamin D (µg)^b^	6.78	5.67	7.33	5.45	0.10	0.96 (0.92–1.01)
Dietary vitamin D (µg)^b^	3.30	1.87	3.69	2.21	0.04	0.88 (0.77–1.00)
Vitamin D supplement (µg)	3.48	5.10	3.64	5.02	0.32	0.97 (0.92–1.03)
Total energy intake (MJ)	6.79	1.89	6.70	1.96	0.72	1.00 (1.00–1.00)
Carbohydrate (g)^b^	209	34.8	204	33.7	0.11	1.01 (1.00–1.01)
Carbohydrate (% energy)	53.7	7.93	52.8	7.55	0.46	1.01 (0.98–1.05)
Protein (g)^b^	54.1	10.1	58.0	11.7	<0.01	0.97 (0.95–0.99)
Protein (% energy)	13.9	2.60	15.0	2.95	<0.01	0.86 (0.78–0.94)
Fat (g)^b^	55.8	11.4	55.6	10.9	0.32	1.01 (0.99–1.03)
Fat (% energy)	32.4	6.88	32.3	6.21	0.63	1.00 (0.97–1.04)
Soybean (g)	82.5	98.4	84.8	82.0	0.71	1.00 (0.99–1.02)
Alcohol (g)	1.73	9.64	0.57	1.88	0.17	1.06 (0.97–1.16)
Dietary calcium (mg)^b^	421	149	440	157	0.06	1.00 (1.00–1.00)
Calcium supplement (mg)	83.6	139	84.7	133	0.19	1.00 (1.00–1.00)
Total calcium (mg)^b^	504	221	524	220	0.03	1.00 (1.00–1.00)

SD, standard deviation; OR, odds ratio; CI, confidence interval.

^a^Cases and controls were compared by using conditional logistic regression analysis. *P* values are adjusted for age (<40, 40–45, 46–50, 51–55, 56–60, ≥60 y), education (junior high school or less, senior high school, some college), parity (0, 1, 2, ≥3 times), use of hormone replacement therapy (yes, no), body mass index (<18.5, 18.5–23.9, 24.0–26.9, ≥27 kg/m^2^), and total energy intake.

^b^Nutrient intakes are energy-adjusted (residuals method).

Fish and eggs provided almost 80% of total dietary vitamin D in both groups (Table 3). Although protein intake was lower in cases than in controls (54.1 vs 58.0 grams per day; Table 2), only milk protein intake was significantly lower in cases (3.47 vs 3.78 grams per day; data not shown).

**Table 3.** Average daily vitamin D intake from food in cases and controls

Foods	Cases (*n* = 200)					Controls (*n* = 200)				
	
	Weight (g)		Vitamin D (µg)		Contribution %	Weight (g)		Vitamin D (µg)		Contribution %
			
	Mean	SD	Mean	SD		Mean	SD	Mean	SD	
Fish other than deep sea fish	21.0	24.1	1.61	1.85	48.9	21.5	22.5	1.65	1.72	44.8
Deep sea fish	10.4	15.0	0.53	0.76	16.1	13.3	20.7	0.68	0.73	18.4
Eggs	24.6	18.8	0.51	0.39	15.5	25.3	20.7	0.52	0.43	14.2
Milk	94.6	112	0.41	0.49	12.6	101	111	0.44	0.48	12.1
Other seafood	9.78	12.6	0.22	0.28	6.8	11.8	13.2	0.27	0.30	7.3
Poultry	17.4	16.7	0.06	0.06	2.0	21.3	23.6	0.07	0.08	2.2
Pork, beef, and lamb	29.9	27.5	0.07	0.06	2.2	34.1	27.7	0.08	0.06	2.2
Light-colored vegetables	106	71.2	0.02	0.01	0.7	94.3	63.8	0.02	0.01	0.5
Liver	1.15	2.66	0.01	0.02	0.3	1.74	4.73	0.01	0.04	0.4
Other organ meats	1.32	2.74	0.01	0.02	0.3	1.83	3.65	0.01	0.04	0.4
Other dairy products^a^	6.54	14.8	0.01	0.02	0.3	7.53	15.5	0.01	0.02	0.3

SD, standard deviation.

^a^Yogurt and cheese.

After controlling for potential confounders, the OR for BC (0.48; 95% CI, 0.24–0.97) was significantly lower in women in the highest quartile of dietary vitamin D intake than in those whose intake was in the lowest quartile. In addition, there was a significant linear trend (*P* = 0.03). After further adjustment for plasma homocysteine, both the difference between Q4 (OR, 0.57; 95% CI, 0.28–1.19) and Q1 and the linear trend were no longer significant. Regarding total vitamin D intake (median intake, 5.20 µg per day), the multivariable ORs (95% CI) across quartiles were 1.00 (referent), 0.51 (0.25–1.02), 0.60 (0.30–1.18), and 0.48 (0.24–0.98); the overall trend was not significant (*P* = 0.10; Table 4).

**Table 4.** Odds ratios and 95% confidence intervals for breast cancer by vitamin D intake

	Quartile of vitamin D intake				*P* for trend^a^
	
	Q1	Q2	Q3	Q4	
Dietary vitamin D^b^, median (range)	1.39 (0.17–1.99)	2.60 (2.00–3.14)	3.83 (3.15–4.94)	6.40 (5.05–10.7)	
No. of cases/controls	46/40	44/39	38/42	30/47	
Model 1^c^	1.00	0.99 (0.50–1.96)	0.74 (0.38–1.45)	0.48 (0.24–0.97)	0.03
Model 2^d^	1.00	1.03 (0.51–2.09)	0.81 (0.41–1.63)	0.57 (0.28–1.19)	0.09

Total vitamin D^b^, median (range)	2.08 (0.17–3.14)	4.26 (3.15–5.55)	7.15 (5.59–10.5)	14.2 (10.7–28.7)	
No. of cases/controls	55/41	31/43	32/41	41/43	
Model 1^c^	1.00 (referent)	0.51 (0.25–1.02)	0.60 (0.30–1.18)	0.48 (0.24–0.98)	0.10
Model 2^d^	1.00 (referent)	0.51 (0.25–1.05)	0.69 (0.34–1.39)	0.52 (0.25–1.07)	0.17

^a^Estimated by entering the median of each quartile as a continuous variable in the model and tested using the Wald test.

^b^Micrograms per day (energy-adjusted; residuals method).

^c^Logistic regression model, adjusted for age (<40, 40–45, 46–50, 51–55, 56–60, ≥60 y), education (junior high school or below, senior high school, some college), parity (0, 1, 2, ≥3 times), use of hormone replacement therapy (yes, no), body mass index (<18.5, 18.5–23.9, 24.0–26.9, ≥27 kg/m^2^), total energy intake, sunlight exposure (<30, 30–59, ≥60 minutes per day), and menopausal status (pre-, post-menopausal).

^d^Logistic regression model adjusted for same covariates as model 1 plus homocysteine (<5.80, 5.80–6.95, 6.96–8.30, ≥8.40 nmol/mL).

The synergistic effect of sunlight exposure and vitamin D intake was not significant (*P* = 0.61). However, a lower risk (OR, 0.47; 95% CI, 0.20–1.09) was found for women who had high levels of both vitamin D intake (≥5 µg per day) and sunlight exposure (>30 min/d), as compared with those who had a lower intake and less sunlight exposure, although the association was not significant (data not shown).

The protective effect of vitamin D against BC that was observed in model 1 was lost after adjustment for protein intake. Stratification of calcium and protein intakes by fish consumption did not alter this finding. Similar findings were seen with total vitamin D intake (data not shown).

The protective link between vitamin D intake and BC was clearer in premenopausal women (Q4 vs Q1: OR, 0.38; 95% CI, 0.14–0.98; *P* for trend = 0.02). Stratification by BMI revealed an association between vitamin D intake and BC in women with a lower BMI (Q4 vs Q1: OR, 0.27; 95% CI, 0.11–0.62; *P* for trend = 0.002). There was a threshold effect of vitamin D intake among subjects with a normal BMI (Q2–Q4 vs Q1: OR, 0.46; 95% CI, 0.23–0.90). Stratification by sunlight exposure did not reveal a significant benefit (Table 5). In subgroup analyses, vitamin D intake in the highest quartile was protective against BC (Q4 vs Q1: OR, 0.35; 95% CI, 0.13–0.94; *P* for trend = 0.039) only in premenopausal women of normal weight (data not shown).

**Table 5.** Odds ratios and 95% confidence intervals for breast cancer associated with vitamin D intake, by menopausal status, body mass index (BMI), and sunlight exposure^a^

	Menopausal status				BMI (kg/m^2^)				Sunlight exposure (minutes per day)			
			
	Cases/ controls	Pre-	Cases/ controls	Post-	Cases/ controls	<24	Cases/ controls	≥24	Cases/ controls	≤30	Cases/ controls	>30
Dietary vitamin D												
Q1	25/32	1.00	23/25	1.00	30/39	1.00	18/18	1.00	22/24	1.00	18/22	1.00
Q2	26/27	1.08 (0.43–2.71)	24/26	0.64 (0.21–2.01)	31/29	0.67 (0.29–1.57)	19/24	2.64 (0.69–10.1)	20/23	0.92 (0.34–2.49)	19/21	0.75 (0.25–2.23)
Q3	29/32	0.69 (0.29–1.61)	22/21	0.86 (0.28–2.66)	37/40	0.55 (0.25–1.22)	14/13	1.19 (0.28–5.06)	19/19	0.94 (0.35–2.55)	23/19	0.73 (0.26–2.06)
Q4	27/16	0.38 (0.14–0.98)	24/21	0.60 (0.20–1.79)	37/20	0.27 (0.11–0.62)	14/17	2.34 (0.51–10.7)	21/18	0.70 (0.26–1.90)	26/12	0.36 (0.12–1.13)
*P* for trend		0.02		0.46		0.002		0.46		0.48		0.08
Q2–Q4 vs Q1		0.67 (0.32–1.37)		0.69 (0.28–1.71)		0.46 (0.23–0.90)		1.97 (0.64–6.05)		0.84 (0.38–1.88)		0.60 (0.25–1.44)

Total vitamin D												
Q1	30/42	1.00	20/25	1.00	30/42	1.00	20/25	1.00	22/30	1.00	19/25	1.00
Q2	32/33	0.44 (0.19–1.01)	17/15	0.81 (0.22–2.96)	35/35	0.51 (0.23–1.15)	14/13	0.35 (0.08–1.58)	20/15	0.51 (0.18–1.45)	23/16	0.65 (0.23–1.88)
Q3	27/16	0.36 (0.15–0.88)	23/22	1.35 (0.42–4.34)	33/22	0.47 (0.21–1.07)	17/16	1.65 (0.38–7.13)	17/17	0.81 (0.29–2.28)	24/15	0.55 (0.19–1.60)
Q4	18/16	0.47 (0.18–1.23)	33/31	0.68 (0.23–1.97)	37/29	0.38 (0.17–0.89)	14/18	1.21 (0.23–6.37)	23/22	0.55 (0.20–1.49)	20/18	0.39 (0.12–1.26)
*P* for trend		0.16		0.43		0.05		0.61		0.38		0.13
Q2–Q4 vs Q1		0.42 (0.21–0.83)		0.87 (0.35–2.21)		0.45 (0.24–0.88)		0.81 (0.26–2.53)		0.61 (0.27–1.37)		0.53 (0.22–1.28)

^a^Logistic regression model adjusted for all the following variables except the stratifying variable: age (<40, 40–45, 46–50, 51–55, 56–60, ≥60 y), education (junior high school or below, senior high school, some college), parity (0, 1, 2, ≥3 times), use of hormone replacement therapy (yes, no), BMI (<18.5, 18.5–23.9, 24.0–26.9, ≥27 kg/m^2^), total energy intake, and sunlight exposure (<30, 30–59, ≥60 minutes per day).

## DISCUSSION

In the present study, dietary vitamin D intake was protective against breast cancer, independent of sunlight exposure, in premenopausal Chinese women of normal weight in northern, subtropical Taiwan. These findings were not dependent on alcohol intake, which was low in this population, or soy intake, which was examined in relation to menopausal status (data not shown).

Our controls were selected from attendees of health examinations that were not covered by the National Health Insurance system. This may mean that controls were more health conscious than cases, which might account for some of the difference in BC incidence. However, there was no difference in family history of breast or ovarian cancer, an important basis of health awareness. Employer-provided financial support for health checkups, rather than health-seeking behavior, might have skewed the control population toward BC protection. Nevertheless, both cases and controls were recruited from the same hospital, and the controls constituted a study base of individuals who would have been diagnosed at the hospital had they developed BC. Indeed, cases were sometimes identified through these same clinics. Therefore, it is unlikely that our findings were biased by selection of controls in regard to their health concerns. In addition, the fact that the controls were younger and better educated has more to do with the availability of the health checkup service than with substantial differences in the population. In any case, when cases and controls were compared by menopausal status, these differences were less apparent (data not shown).

Because the controls were younger than the cases, possibly due to a cohort effect, some reproductive risk factors were atypical in the crude analyses. In white women, higher parity is protective for BC. In our study population, the transition in BC risk occurs principally in premenopausal women, in whom the risk factors are different. Our matching protocol may have resulted in a situation where cases were more likely to be multiparous; however, a study of premenopausal Chinese Singaporeans found that BC risk was not associated with parity.^25^ Nevertheless, in all analyses, we adjusted for parity, in addition to age, education, and HRT.

There is also the possibility of recall bias. However, in our study, vitamin D intake was derived from food and supplements whose vitamin D content was unknown to the subjects. Indeed, when the data were collected, people were not generally aware that vitamin D and sunlight exposure might be related to BC.

That vitamin D may protect against cancer is of particular interest in regard to BC,^26^^,^^27^ beyond its immediate vitamin D receptor-mediated role in cellular differentiation.^28^^,^^29^ The mechanisms of interest need to be investigated in Northeast Asian women, who are generally of shorter stature than whites but are now increasing in height, have a diet that includes rice, greens, soy, pork, and fish, and live in the subtropics but have an aversion to direct sunlight. First, vitamin D is important in growth, which itself may contribute to BC risk in later life^27^; tallness is a risk factor for BC,^6^ and adequate vitamin D may encourage attainment of optimal height. Secondly, body fat is a likely risk factor for BC,^4^ and fish liver oil, which contains vitamin D_3_, may protect against both overweight in adolescence^30^ and the formation of adipocytes with altered vitamin D bioavailability.^31^ Thirdly, dietary vitamin D is often accompanied by other potentially cancer-protective substances, like vitamin A and omega-3 fatty acids. Finally, a favorable vitamin D status may reflect sunlight exposure due to an outdoor lifestyle.

Vitamin D deficiency would appear to be unlikely in subtropical northern Taiwan because the duration of daylight exceeds 11 hours throughout the year. However, if a subpopulation sought to avoid sunlight exposure in such areas, there would be a wide range of UV-dependent skin synthesis of vitamin D. Our study showed that vitamin D intake may protect against BC in premenopausal Taiwanese who are not overweight (BMI <24), especially if they have limited sunlight exposure (≤30 minutes per day). This means that vitamin D status is more important in younger women of normal weight, which is consistent with studies that show a higher risk for BC in premenopausal women with low body fat.^2^

One explanation for the protective effect of body fat against premenopausal BC is that sex hormone-binding globulin levels are elevated, and free sex hormones levels are lower, in women with a higher BMI.^32^ The relevance of this to premenopausal BC risk may depend on breast estrogen-receptor positivity. Insulin-like growth factor (IGF) level and body size have also been linked to premenopausal BC.^33^ IGF is modulated by levels of IGF binding protein (IGFBP) and various cytokines, especially adiponectin.^34^ Vitamin D might increase levels of IGFBP^35^ and adiponectin,^36^ both of which may protect against BC.^34^ In premenopausal women with low body fatness, vitamin D may protect against BC in several ways.^37^ There may also be unique vitamin D receptor polymorphisms relevant to BC risk in Taiwanese women.^38^

We found that while the combined vitamin D intake from food and supplements was protective, intake from supplements alone was not. However, this negative finding for supplements may be a result of the limited sample size in regard to supplement use and/or heterogeneity in supplement use (D_2_ or D_3_). Caution must thus be exercised in extrapolating the present dietary findings.

Protein intake was protective against BC, and adjustment for protein intake made the relative risk associated with vitamin D nonsignificant. However, when adjustment was limited to non-fish protein, the protective effect of vitamin D remained. This suggests that vitamin D protection is mainly from consumption of aquatic animals (fin fish). It is also possible that consumption of soy and phytoestrogens partially explains the protective effect of protein in Taiwan, but such an effect was not detectable.^39^

Adjustment for calcium intake, a partial indicator of dairy intake, did not diminish the protective effect of vitamin D (data not shown). Although calcium and vitamin D might synergistically protect against BC, the evidence for such an effect is marginal. In a US study, high calcium and vitamin D intakes resulted in moderate protection in premenopausal women, and a statistical interaction between these 2 nutrients was observed in postmenopausal women.^12^ A study in Shanghai, China showed an inverse relation between BC and calcium intake, and a significant trend when the calcium came from poultry consumption.^40^ Poultry skin can be a significant source of vitamin D in the Chinese diet, which may confound this finding. In Taiwan, dairy products are not fortified with vitamin D, with the exception of imported powdered milk, which is consumed mainly by older women.^41^

A meta-analysis of diet, physical activity, and BC by the World Cancer Research Fund/American Institute for Cancer Research^42^ showed that diet, physical activity, and low body fatness were risk factors in premenopausal women. Chie et al found that a higher BMI was protective against BC in premenopausal Taiwanese.^43^ This is relevant to our findings, which suggest that vitamin D is protective against BC in such women. However, physical activity did not contribute to the difference in susceptibility to BC in cases and controls in our study, although, once again, this may be due to the small sample size.

The population attributable risk percentages (PAR%) were 10%, 11.6%, and 27.5% for dietary vitamin D intakes of 3.18, 3.33, and 5 µg per day, respectively (data not shown). Thus, a daily vitamin D intake of 5 µg appears to be critical in BC protection in premenopausal women. This is the DRI in Taiwan and it may need to be increased, provided it is food-based. The median intake for the highest quartile of the present subjects, ie, those who had the highest level of protection against BC (Table 4), was 6.40 µg per day (range, 5.05–10.7). Intake of vitamin D had a greater positive impact on BC risk when sunlight exposure was higher (data not shown). Because the use of a FFQ could result in systematic error in the estimation of intakes, caution is advised when interpreting these values.

The PAR% for sunlight exposure (≤30 vs >30 minutes per day) was 8.51%, although it was not a determinant of BC. Greater sunlight exposure might be beneficial, but this recommendation is likely to conflict with cultural preferences and growing global concern regarding skin cancer in an era of climate change. UV exposure increases the risk of skin cancer, and this needs to be factored into the overall risk-benefit equation. Fortunately, skin cancer incidence in Taiwan is relatively low. Further guidance regarding the optimal vitamin D status for premenopausal women comes from a German study in which the risk for BC declined to a plateau at a plasma 25-hydroxyvitamin D level of approximately 75 nmol/L, which may not be achievable by diet alone.^44^

Several limitations of this study warrant mention. Vitamin D intake was assessed by a short FFQ, which was not validated against other diet assessment methods. However, vitamin D is found in very few foods and our FFQ included all of these, in the form in which they are consumed in Taiwan. In addition, we validated folate and vitamin B-6 intakes against blood measurements. The Spearman rank correlation coefficients were 0.34 and 0.31, respectively, which indicates that our dietary methodology has relatively good predictive power, although the variance for such relationships does not depend only on food intake. In addition, there are limited data on vitamin D food composition in Taiwan. Thus, the accuracy of estimates of vitamin D intake may be compromised. Nevertheless, the ranking of this vitamin is likely to be valid. This is supported by the findings of our previous population-based study of adult women whose diets were assessed by 24-hour recall. Intakes were similar to those of the present study in terms of quantity (3.24–6 µg per day for various age groups) and food source.^41^

Given the small sample, some of the findings in stratified analyses might be due to chance. However, the observation that vitamin D protects against BC in premenopausal women is still significant. Our negative findings in postmenopausal women will need to be re-examined in a larger population.

The geographic and demographic characteristics of this population present both an advantage and a limitation. Breast cancer incidence in Taiwan has been low, but is now increasing, which makes it more of a transitional phenomenon than in Western countries. Moreover, as compared with Western countries, breast cancer is seen more often in younger women. Fortunately, it is premenopausal women of normal weight who appear to benefit from higher vitamin D intake. Although we have made unique observations, extrapolation to other populations needs to be circumspect. It does, however, appear likely that a subtropical location with ample sunlight does not provide complete protection against vitamin D-related disorders, including breast cancer. We hope that our investigation increases interest in vitamin D status in Asians, and Chinese in particular, both with respect to policy and future research.
